# Swedish SDQ Norms for Internalizing and Externalizing Problems in Preschoolers

**DOI:** 10.1111/sjop.70124

**Published:** 2026-06-17

**Authors:** Anton Dahlberg, Anna Sarkadi, Raziye Salari

**Affiliations:** ^1^ Child Health and Parenting (CHAP), Department of Public Health and Caring Sciences Uppsala University Uppsala Sweden; ^2^ Department of Clinical Psychology and Psychotherapy Friedrich‐Alexander‐Universität Erlangen‐Nürnberg Erlangen Germany

**Keywords:** mental health, norms, preschool children, psychometrics, strengths and difficulties questionnaire, Sweden

## Abstract

The Strengths and Difficulties Questionnaire (SDQ) is one of the most widely used instruments for assessing mental health in children. While Swedish norms for the SDQ have previously been published for children aged 3–5 years, percentile‐based cutoffs for the broader internalizing and externalizing scales are still lacking. This study aimed to extend existing Swedish SDQ norms by providing population‐based cutoffs for the composite internalizing and externalizing scales in preschool children. Data were derived from a large, population‐based sample of children aged 3–5 years in Uppsala County, including ratings from both parents and preschool teachers. Internalizing and externalizing scores were computed by summing the relevant SDQ subscales, and percentile cutoffs were calculated by age, gender, and rater, with the 80th and 90th percentiles used to indicate borderline and abnormal levels of difficulties. Cutoff values were consistently lower in teacher ratings than in parent ratings, and across most age groups and scales girls had lower cutoff values than boys. Age variation was modest, with a general tendency towards slightly lower cutoff scores with increasing age. These new normative cutoffs extend the Swedish SDQ norms for preschoolers to include the widely used composite scales and support more comprehensive use of the SDQ in both clinical screening and research on young children's mental health.

## Key Points

- This study provides the first Swedish population‐based, percentile‐derived cutoffs for the SDQ internalizing and externalizing composite scales in preschool children aged 3–5 years.
- Cutoffs are presented separately by age, gender, and rater (parent and preschool teacher), based on a sample of 12,245 children.
- Teacher ratings yielded consistently lower cutoff values than parent ratings, underscoring the need for rater‐specific thresholds.
- Girls generally had lower cutoffs than boys, and scores tended to decrease slightly with age.
- The norms complement existing Swedish SDQ norms and support broader use of the composite scales in clinical screening and research.

## 1 Introduction

Since its development in the 1990s, the Strengths and Difficulties Questionnaire (SDQ) has become one of the most widely used tools for identifying emotional and behavioral difficulties in children (Goodman 1997, 2001). It is employed globally in both clinical settings and population‐based research (Stone et al. 2010), with thousands of scientific articles published on or using the questionnaire (http://www.sdqinfo.org).

The SDQ comprises 25 questions divided into five subscales: emotional symptoms, conduct problems, hyperactivity/inattention, peer problems, and prosocial behavior. Each item is rated on a 3‐point scale (0–2), with each subscale consisting of five summed item scores (subscale score range 0–10). The four difficulty subscales (all subscales save prosocial behavior) can be summed to produce a total difficulties score (range 0–40).

The psychometric properties of the SDQ have been evaluated in many studies across the globe, with generally favorable results in many studies (e.g., Dahlberg et al. 2018; Klein et al. 2013; Ortuño‐Sierra et al. 2015). However, researchers have also raised concerns regarding the SDQ's construct validity. This is especially true for the self‐rated version of the questionnaire, aimed towards older children and youth, where the overall factor structure and subscales such as the peer problems subscale have shown poor fit with the overall factor model (Aarø et al. 2022).

The SDQ can be used with children in preschool age, a developmental period characterized by rapid changes in emotional reactivity, emotion regulation, and social behavior (Cole et al. 2004; Gross 2013; Kopp 1989). During this period, children gradually acquire the capacity to identify, express, and regulate emotions, while still relying heavily on external support from significant adults (Kopp 1989; Thompson 2014). Transient fears, worries, social withdrawal, and emotional dysregulation are therefore common and often represent normative aspects of development rather than indicators of psychopathology (Carter et al. 2004; Egger and Angold 2006; Wakschlag et al. 2010).

This developmental context makes it particularly challenging to determine when emotional and behavioral expressions exceed what can be considered age‐appropriate variation and instead reflect clinically relevant internalizing problems. Symptoms such as anxiety, sadness, or peer‐related difficulties may fluctuate with developmental stage, situational demands, and environmental context, and may not yet show the stability typically required for clinical diagnosis (Carter et al. 2004; Dougherty et al. 2013). Consequently, the distinction between normal development and early manifestations of mental health difficulties can be difficult to determine in preschool populations (Egger and Angold 2006; Wakschlag et al. 2010). At the same time, evidence of psychopathology can be seen in early childhood (Egger and Angold 2006), and timely assessment and monitoring of emerging emotional and behavioral problems is therefore warranted.

To support interpretation in this developmental period, in Sweden, normative data for the SDQ in preschool‐aged children have previously been published (Björnsdotter et al. 2013; Dahlberg et al. 2020; Gustafsson et al. 2017), including cutoffs for the total difficulties score and the five original subscales. Dahlberg et al. (2020) presented Swedish normative data for the SDQ among children aged 3–5 years, based on ratings from both parents and preschool teachers. The study provided age‐, gender‐, and rater‐specific percentile cutoffs for each of the original SDQ subscales as well as for the total difficulties score. These norms have been applied in several studies, assessing the relationship between SDQ scores and factors such as subsequent mental health problems and socio‐economic deprivation (Durbeej et al. 2024; Nahar et al. 2025).

In addition to the total score and the five original subscales, the SDQ can be summarized into two broader dimensions: internalizing problems and externalizing problems. Internalizing problems refer to inwardly directed difficulties that manifest as emotional distress within the individual, including symptoms such as anxiety, depression, social withdrawal, and somatic complaints (Achenbach 1991; Forns et al. 2011). In contrast, externalizing problems are outwardly directed behaviors characterized by impulsivity, aggression, noncompliance, and rule‐breaking, which cause conflict in the external environment (Achenbach 1991; Forns et al. 2011). These two empirically derived broad‐band dimensions, originally proposed by Achenbach, capture the predominant emotional (internalizing) and behavioral (externalizing) domains of child and adolescent psychopathology (Achenbach et al. 2016).

In the SDQ, internalizing problems comprise the emotional symptoms and peer problems subscales, whereas externalizing problems comprise the conduct problems and hyperactivity/inattention subscales (Goodman et al. 2010). These composite scales are frequently used in both clinical screening and population studies to capture a more general overview of psychological difficulties. Empirical studies have shown that these broad‐band scale scores may have better discriminant validity than individual subscales in low‐symptom populations (e.g., Carneiro et al. 2023; Goodman et al. 2010). However, no corresponding norms for Swedish preschool children have yet been provided for the broader subscales.

Against this background, empirically derived, age‐ and rater‐specific normative cutoffs are essential for the interpretation of screening instruments such as the SDQ (Kremer et al. 2015; Woerner et al. 2004). Because behavioral and emotional scores change systematically across the preschool years as a function of normal development, applying the same cutoffs across age groups risks misclassifying children whose scores simply reflect age‐typical functioning rather than clinically meaningful deviation (Dahlberg et al. 2020; Janitza et al. 2020; Maurice‐Stam et al. 2018). Similarly, given documented differences between parent and teacher ratings, rater‐specific cutoffs are necessary for valid interpretation across informants (De Los Reyes and Kazdin 2006; Fält et al. 2018; Gustafsson et al. 2016). Norm‐based thresholds do not imply pathology per se, but provide a structured way to identify children whose emotional or behavioral functioning deviates markedly from that of their peers, and who may therefore benefit from closer monitoring or further assessment. At present, there are no published norms for the internalizing and externalizing subscales of the SDQ for preschool aged children in Sweden.

### 1.1 Aims

The present study aimed to extend existing Swedish SDQ norms for preschool children by providing population‐based, percentile‐derived cutoffs for the internalizing and externalizing composite scales, presented separately by age (3, 4, and 5 years), gender, and rater (parent and preschool teacher).

## 2 Methods

### 2.1 Participants and Procedures

The present study was based on the same dataset and procedures described in Dahlberg et al. (2020). Data were derived from a large, population‐based sample of Swedish preschool children aged 3–5 years, collected between 2013 and 2017, as part of a public health intervention study in Uppsala County (Salari et al. 2013). Parents were invited to complete the SDQ during their child's annual check‐up at child health centers, and were asked to pass a separate questionnaire to the child's preschool teacher for completion. Parent ratings were based on either the father's or mother's SDQ rating. If both the mother's and father's ratings were available for a child, one was selected at random. Priority was given to the parent with more complete data when applicable. After exclusion of questionnaires with insufficient data, duplicate ratings, or ineligible informants, the final analytical sample comprised 11,196 parent‐rated and 9083 preschool teacher–rated SDQs, representing 12,245 unique children. Sociodemographic characteristics of the sample are displayed in Table 1. The sample was skewed towards higher parental education levels: approximately 70% of parents had university‐level education, compared with approximately 50% in the general Swedish population of the same age range (Statistics Sweden; www.scb.se).

**TABLE 1:** Sociodemographic characteristics of children and parents in the study sample.

Sociodemographic variables	3‐year‐olds		4‐year‐olds		5‐year‐olds	
	*n*	%	*n*	%	*n*	%
Child gender						
Girl	1769	49.3	1908	49.5	1813	48.3
Boy	1815	50.6	1946	50.5	1934	51.6
Not specified	6	0.1	2	< 0.1	3	0.1
Parent gender						
Female (mother)	2034	56.7	2170	56.3	2035	54.3
Male (father)	1553	43.3	1684	43.7	1711	45.6
Completed together	3	< 0.1	2	< 0.1	4	0.1
Parental highest level of education^a^						
High school or less	1075	30.5	1090	28.6	1001	27.2
University	2453	69.5	2715	71.4	2673	72.8
Parent country of birth^b^						
Sweden	3008	84.2	3229	84.6	3128	84.8
Other	542	15.8	587	15.4	560	15.2
Parent relationship status^c^						
Single parent	150	4.3	210	5.5	253	6.9
Cohabiting	1275	36.1	1215	32.1	1031	28.1
Married	2052	58.2	2298	60.6	2331	63.5
Live‐apart	43	1.2	53	1.4	44	1.2
Other	7	0.2	13	0.3	12	0.3
Parent age (mean, SD)^d^	36.1	5.4	37.1	5.3	38.2	5.4

^a^ Missing data from 62 3‐year‐olds, 51 4‐year‐olds, and 76 5‐year‐olds.

^b^ Missing data from 40 3‐year‐olds, 40 4‐year‐olds, and 62 5‐year‐olds.

^c^ Missing data from 63 3‐year‐olds, 67 4‐year‐olds, and 79 5‐year‐olds.

^d^ Missing data from 43 3‐year‐olds, 40 4‐year‐olds, and 69 5‐year‐olds.

### 2.2 Measures

The SDQ is a brief, widely used screening instrument for emotional and behavioral problems in children aged 2–17 years (Goodman 1997). It comprises 25 items divided into five subscales: emotional symptoms, conduct problems, hyperactivity/inattention, peer problems, and prosocial behavior. Each item is rated on a three‐point scale (0 = not true, 1 = somewhat true, 2 = certainly true), yielding subscale scores ranging from 0 to 10. The four difficulty subscales can be summed to produce a total difficulties score (range 0–40).

The data were collected within a longitudinal study framework, which allowed individual children to contribute SDQ ratings at one, two, or three time points corresponding to ages 3, 4, and/or 5 years. In the present study, however, analyses were conducted cross‐sectionally within each age group. Each SDQ assessment was analyzed according to the child's age at the time of rating, and no within‐child longitudinal modeling was performed. The purpose of this approach was to derive age‐specific normative cutoffs rather than to examine individual developmental trajectories over time.

### 2.3 Data Analysis

Internalizing and externalizing scores were computed by summing the relevant subscales, following the established operationalization by Goodman et al. (Goodman et al. 2010): internalizing problems = emotional symptoms + peer problems; externalizing problems = conduct problems + hyperactivity/inattention. Both composite scores therefore range from 0 to 20. The psychometric properties of the SDQ in this sample, including factor structure, internal consistency, and measurement invariance across age, gender, and parental education, have been evaluated in detail elsewhere and found to be satisfactory (Dahlberg et al. 2018).

Cutoff values for internalizing and externalizing SDQ scores were calculated separately for each age group and rater, using the 80th and 90th percentiles to indicate borderline and abnormal levels, respectively (Goodman 1997).

## 3 Results

Table 2 comprises cutoff values from the full sample, while gender‐specific cutoffs can be found in Table 3. Mean SDQ scores were significantly lower in teacher ratings compared to parent ratings. This was true for both internalizing problems (*t*_6386.3_ = 16.633, *p* < 0.001 for 3‐year‐olds; *t*_6992.2_ = 21.118, *p* < 0.001 for 4‐year‐olds; *t*_6742.8_ = 21.47, *p* < 0.001 for 5‐year‐olds) and externalizing problems (*t*_6117.2_ = 22.908, *p* < 0.001 for 3‐year‐olds; *t*_6495_ = 20.15, *p* < 0.001 for 4‐year‐olds; *t*_6355.5_ = 18.206, *p* < 0.001 for 5‐year‐olds).

**TABLE 2:** Percentile‐based cutoffs (80th and 90th) for internalizing and externalizing SDQ scales by age and rater.

	Parents		Preschool teachers	
	Borderline	Abnormal	Borderline	Abnormal
3‐year‐olds	*n* = 3590		*n* = 2938	
Internalizing problems	4	5	2	4
Externalizing problems	7	9	5	7
4‐year‐olds	*n* = 3856		*n* = 3145	
Internalizing problems	3	5	2	3
Externalizing problems	7	9	5	7
5‐year‐olds	*n* = 3750		*n* = 3000	
Internalizing problems	3	5	2	3
Externalizing problems	6	8	4	7

**TABLE 3:** Gender‐specific, percentile‐based cutoffs (80th and 90th) for internalizing and externalizing SDQ scales by age and rater.

	Girls				Boys			
	Parents		Preschool teachers		Parents		Preschool teachers	
	Borderline	Abnormal	Borderline	Abnormal	Borderline	Abnormal	Borderline	Abnormal
3‐year‐olds	*n* = 1769		*n* = 1437		*n* = 1815		*n* = 1498	
Internalizing problems	3	5	2	3	4	5	2	4
Externalizing problems	7	9	4	6	8	9	6	8
4‐year‐olds	*n* = 1908		*n* = 1542		*n* = 1946		*n* = 1597	
Internalizing problems	3	4	2	3	4	5	2	3
Externalizing problems	6	8	4	6	7	9	6	9
5‐year‐olds	*n* = 1813		*n* = 1471		*n* = 1934		*n* = 1529	
Internalizing problems	3	4	2	3	3	5	2	3
Externalizing problems	5	7	3	5	7	8	6	8

As can be seen in Table 2, there was relatively little variation in internalizing and externalizing cutoff scores across ages and raters overall. However, a general trend of decreasing cutoff scores with age was observed. This was not evident for all scales, genders, and raters. For instance, the 90th percentile for internalizing problems remained stable across all age groups in parent ratings, and a similar pattern was observed for the teacher‐rated externalizing problems. Girls had lower cutoff values for almost all ages and across scales, as can be seen in Table 3.

## 4 Discussion

The present study provides percentile‐based cutoffs for the internalizing and externalizing SDQ scales in Swedish preschoolers aged 3–5 years. The findings are consistent with those in Dahlberg et al. (2020): cutoff scores decreased slightly with age, parent ratings yielded higher scores than teacher ratings, and girls scored lower than boys in general.

The present norms are fully compatible with previously reported Swedish SDQ norms for this age group (Dahlberg et al. 2020), as they are based on the same sample and follow the same scoring procedures. They provide clinicians and researchers with additional tools for interpreting SDQ scores at both the subscale and composite level, in line with recommendations for when to use broader internalizing and externalizing subscales versus the original five subscales (Goodman et al. 2010).

The modest age‐related differences observed in the present cutoffs should be interpreted in light of the rapid developmental changes that characterize the preschool years (Kopp 1989; Thompson 2014). Emotional reactivity, self‐regulation, and social functioning undergo substantial maturation during this period (Cole et al. 2004; Egger and Angold 2006), which may contribute to both normative variability and age‐related shifts in SDQ score distributions (Dahlberg et al. 2020; Janitza et al. 2020). This further underscores the importance of age‐specific normative reference values when interpreting emotional and behavioral screening results in early childhood.

A key strength of our study is the use of a large, community‐based sample. A primary limitation is that the sample over‐represented parents with higher education (approximately 70% university‐educated vs. approximately 50% nationally), which may limit generalizability to populations with different socioeconomic compositions. Although the sample is large and population‐based, it was drawn exclusively from Uppsala County, which limits geographic representativeness within Sweden. Regional variation in sociodemographic characteristics, service provision, and cultural factors may influence SDQ score distributions, and the extent to which the present norms generalize to other Swedish regions remains unknown.

A further consideration concerns the peer problems subscale, which contributes to the internalizing composite alongside the emotional symptoms subscale. Although the factor structure of the SDQ, including the peer problems subscale, showed acceptable fit and satisfactory psychometric properties in this sample (Dahlberg et al. 2019), the peer problems subscale has been noted as psychometrically heterogeneous in other populations and age groups (Aarø et al. 2022; Carneiro et al. 2023; Forns et al. 2011). Its conceptual overlap with internalizing difficulties may also vary by developmental stage and informant, which should be taken into account when interpreting internalizing composite scores in preschool‐aged children.

A further consideration is that the data underlying the present norms were collected approximately a decade ago. Normative data for widely used screening instruments such as the SDQ are, however, typically updated at relatively long intervals, and many existing national norms are based on data of similar or greater age. Percentile‐based cutoffs reflect the relative distribution of scores within a population rather than fixed clinical criteria. As such, they are commonly used in population‐based screening to facilitate interpretation across contexts and time periods, although secular changes in symptom levels may still influence normative values. Accordingly, the present cutoffs should be viewed as normative reference values intended to aid interpretation of SDQ scores in preschool children, rather than as definitive indicators of psychopathology. Future studies may nonetheless wish to examine whether updated norms are warranted as population characteristics and contextual factors change over time.

The internalizing and externalizing composites used in the present study follow an established SDQ operationalization and are widely applied in clinical screening and epidemiological research, as well as for assessments in low‐risk populations (Achenbach et al. 2012; Elberling et al. 2010); see also www.sdqinfo.org. As aggregated constructs, they are particularly useful for early screening, longitudinal follow‐ups, and the evaluation of interventions at a more general level (Achenbach et al. 2016; Goodman et al. 2010). At the same time, the peer problems subscale is known to be heterogeneous, and its contribution to broad‐band constructs may vary by developmental stage, informant, and context (Aarø et al. 2022; Carneiro et al. 2023; Forns et al. 2011). In preschool children, peer‐related difficulties such as social withdrawal and limited peer engagement are commonly conceptualized as internalizing expressions, although this interpretation may differ at older ages or in self‐report data (Egger and Angold 2006; Wakschlag et al. 2010).

Future research should examine the clinical and predictive utility of these new cutoffs for internalizing and externalizing problems. It remains to be established whether the percentile thresholds derived here effectively identify children at risk for later mental health difficulties or in need of early intervention. Evaluating the practical application of these norms in both clinical and public health contexts would help determine their value beyond descriptive use.

## 5 Conclusion

This study extends the Swedish SDQ norms for preschool children by providing population‐based cutoffs for the widely used internalizing and externalizing composite scales. While earlier norms have covered the original five subscales and the total difficulties score, no corresponding normative values have previously been available for these broader composites in Swedish preschool‐aged populations.

The availability of age‐, gender‐, and rater‐specific cutoffs for the internalizing and externalizing scales facilitates more flexible and comprehensive interpretation of SDQ results in both research and clinical screening contexts, particularly in epidemiological studies and longitudinal follow‐ups where broad‐band measures are commonly applied.

The present cutoffs are intended as normative reference values to support the interpretation of SDQ scores at the group and individual screening level and should not be interpreted as diagnostic thresholds or as substitutes for clinical assessment.

## Author Contributions

**Anton Dahlberg:** conceptualization, data curation, statistical analysis, writing – original draft, writing – review and editing. **Anna Sarkadi:** conceptualization, writing – review and editing. **Raziye Salari:** conceptualization, data curation, statistical analysis, writing – review and editing.

## Funding

This work was supported by Joint grant from the Swedish Research Council, the Swedish Research Council for Sustainable Development, the Swedish Council for Working Life and Social Research, and VINNOVA (259‐2012‐68), and Forskningsrådet om Hälsa, Arbetsliv och Välfärd (2021‐01046).

## Ethics Statement

The study was approved by the Regional Ethical Review Board in Uppsala (Dnr 2012/437).

## Consent

Informed consent was obtained from all participants included in the study.

## Data Availability

The data that support the findings of this study are available on request from the corresponding author. The data are not publicly available due to privacy or ethical restrictions.
